# Resistance characterization of hepatitis C virus genotype 2 from Japanese patients treated with ombitasvir and paritaprevir/ritonavir

**DOI:** 10.1002/jmv.24923

**Published:** 2017-09-22

**Authors:** Gretja Schnell, Rakesh Tripathi, Preethi Krishnan, Jill Beyer, Thomas Reisch, Michelle Irvin, Tatyana Dekhtyar, Carolyn Setze, Lino Rodrigues‐Jr, Katia Alves, Margaret Burroughs, Rebecca Redman, Kazuaki Chayama, Hiromitsu Kumada, Christine Collins, Tami Pilot‐Matias

**Affiliations:** ^1^ Research & Development AbbVie Inc. North Chicago Illinois; ^2^ Department of Gastroenterology and Metabolism Hiroshima University Hiroshima Japan; ^3^ Department of Hepatology Toranomon Hospital Tokyo Japan

**Keywords:** genotype 2, hepatitis C virus, ombitasvir, paritaprevir

## Abstract

Treatment of HCV genotype (GT) 2‐infected Japanese patients with paritaprevir (NS3/4A inhibitor boosted with ritonavir) and ombitasvir (NS5A inhibitor) without ribavirin for 12 weeks in the phase 2 study M12‐536, and with ribavirin for 16 weeks in phase 3 study GIFT II resulted in SVR rates of 72.2% to 91.5%. Overall, 11 out of 125 patients with GT2a and 37 out of 79 patients with GT2b infection experienced virologic failure. The prevalence of baseline polymorphisms in NS3 and NS5A and their the impact on treatment outcome, as well as the development of viral resistance in GT2‐infected patients experiencing virologic failure were evaluated by HCV NS3 and NS5A population and clonal sequence analyses. Baseline polymorphisms in NS3 that confer resistance to paritaprevir were rare in both GT2a‐ and GT2b‐infected patients, while baseline polymorphisms in NS5A that confer resistance to ombitasvir were detected in 11.2% and 14.1% of the GT2a‐ and GT2b‐infected patients, respectively. There was no significant impact of baseline polymorphisms on treatment outcome in Japanese patients. The most common treatment‐emergent substitutions at the time of virologic failure occurred at amino acid positions 168 in NS3 and 28 in NS5A in both GT2a‐ and GT2b‐infected patients. Although there was a higher rate of virologic failure in patients with GT2b infection, the resistance analyses presented in this report support the conclusion that testing for baseline resistance‐associated polymorphisms is not warranted for HCV GT2‐infected patients treated with a regimen of ombitasvir/paritaprevir/ritonavir + ribavirin for 16 weeks.

## 1 INTRODUCTION

Hepatitis C virus (HCV) infection is a leading cause of hepatocellular carcinoma (HCC) and chronic liver disease, with approximately 185 million people infected worldwide.^1^ HCV is genetically diverse and classified into 7 confirmed genotypes and 67 subtypes,^2^ of which genotype (GT) 2 accounts for 9% of global infections.^3^ Japan has a high prevalence of HCV GT2 infection representing 30% of HCV‐infected patients, of which two‐thirds are infected with subtype 2a and one‐third are infected with subtype 2b.^4^ The majority of the remaining 70% of HCV infections in Japan are caused by GT1b.^4^ The genotype distribution of HCV infections in Japan has changed over time, and recent studies have reported a decrease in GT1 prevalence and an increase in GT2 prevalence in the HCV‐infected patient population which was correlated with differences in transmission routes, especially for individuals born after 1970 where <50% of infections were reported as GT1b.^4^, ^5^ Given that approximately 70% of HCC cases are caused by HCV in Japan, and that HCC prevalence has increased over the past 50 years,^4^, ^6^ direct‐acting antiviral (DAA) therapies are needed to treat both HCV GT1 and GT2 infections in Japan.

Several DAA‐based therapies with high sustained virologic response (SVR) rates at post‐treatment week 12 (SVR_12_) are approved for the treatment of HCV GT1 infection in Japan.^7^, ^8^, ^9^, ^10^ For HCV GT2 infection, the standard of care was previously a combination of pegylated interferon alpha (pegIFN) plus ribavirin (RBV) for 24 weeks, which demonstrated SVR rates of 71‐80% in large clinical trials worldwide.^11^, ^12^, ^13^ The first IFN‐free DAA regimen for HCV GT2 infection with high SVR_12_ rates of 93‐98% was the combination of the NS5B nucleotide polymerase inhibitor sofosbuvir (SOF) plus RBV for 12 weeks,^14^, ^15^ approved for the treatment of GT2‐infected patients in 2015.^16^ A treatment regimen of ombitasvir/paritaprevir/ritonavir (OBV/PTV/r) plus RBV for 16 weeks was recently approved for the treatment of GT2‐infected patients in Japan.

Ombitasvir is an HCV NS5A inhibitor^17^ and paritaprevir is an HCV NS3/4A inhibitor (identified by AbbVie and Enanta) that is co‐administered with the pharmacokinetic enhancer ritonavir.^18^ Ombitasvir and paritaprevir have demonstrated in vitro antiviral activity against multiple HCV genotypes, with 50% effective concentration (EC_50_) values of 12 and 4.3 pM for ombitasvir against GT2a and GT2b,^19^ respectively, and EC_50_ values of 9.8 and 107 nM for paritaprevir against GT2a and GT2b, respectively. A dose‐ranging Japanese phase 2 study (M12‐536) reported an SVR_24_ rate of 72.2% in treatment‐experienced HCV GT2‐infected Japanese patients without cirrhosis who received OBV/PTV/r dosed at 25/150/100 mg for 12 weeks.^7^ Due to the lower overall SVR_24_ rate for the treatment of HCV GT2b infection in study M12‐536, extended treatment duration of 16 weeks and the addition of RBV to the treatment regimen were evaluated in phase 3 study GIFT‐II. The Japanese GIFT‐II study reported SVR_12_ rates of 91.5% and 75.0%, respectively, in treatment‐naïve and treatment‐experienced HCV GT2‐infected Japanese patients without cirrhosis who received co‐formulated OBV/PTV/r plus RBV for 16 weeks.^20^ The current report was designed as a comprehensive clinical virology resistance analysis for GT2‐infected Japanese patients enrolled in clinical studies M12‐536 or GIFT‐II. This analysis examined the GT2 subtype prevalence in M12‐536 and GIFT‐II, assessed the impact of baseline polymorphisms on SVR rates, and analyzed the development and persistence of viral resistance amino acid substitutions in Japanese GT2‐infected patients who experienced virologic failure.

## 2 MATERIALS AND METHODS

### 2.1 Ethics statement

The M12‐536 and GIFT‐II studies were conducted in accordance with guidelines of the International Conference of Harmonization, applicable regulations and guidelines governing clinical study conduct, and ethical principles expressed in the Declaration of Helsinki. The study protocols were approved by the relevant institutional review boards and regulatory agencies, and all patients provided written informed consent.

### 2.2 Study design and patient population

The M12‐536 study (ClinicalTrials.gov identifier: NCT01672983) was a randomized, open‐label, dose‐ and duration‐ranging, phase 2 study that evaluated the safety and efficacy of ombitasvir and paritaprevir/r without RBV in patients with HCV GT 1b or GT2 infection. The study design, safety, and efficacy results through SVR_24_ from patients with HCV GT1b or GT2 infection were previously reported.^7^ The GT2‐infected patient population included non‐cirrhotic, pegIFN/RBV treatment‐experienced patients (null responders, partial responders, and relapsers) in Japan. Patients with GT2 infection were randomized in a 1:1 ratio to receive once‐daily ombitasvir (25 mg) plus either paritaprevir/ritonavir 100/100 mg or 150/100 mg for 12 weeks.

The GIFT‐II study (ClinicalTrials.gov identifier: NCT02023112) was a randomized, open‐label, duration‐finding phase 3 study that evaluated the safety and efficacy of co‐formulated ombitasvir/paritaprevir/ritonavir (25/150/100 mg, QD) plus RBV (weight‐based, 400‐1000 mg BID) administered for 12 or 16 weeks to treatment‐naïve and IFN (alpha, beta, or pegIFN) ± RBV treatment‐experienced HCV GT2‐infected Japanese patients without cirrhosis or with compensated cirrhosis. The study design, safety, and efficacy results through SVR_12_ were previously reported.^20^

### 2.3 HCV genotype and subtype determination by phylogenetic analysis

The Versant HCV Genotype Inno‐LiPA Assay v2.0 (LiPA 2.0) was used to determine HCV genotype for enrollment of patients with chronic HCV GT2 infection for studies M12‐536 and GIFT‐II, but was unable to identify the viral subtype for the majority of GT2‐infected patients. The preliminary viral subtype was therefore determined by phylogenetic analysis of a 329 nucleotide (nt) region of the NS5B gene that was PCR amplified from baseline samples of HCV GT2‐infected patients.^21^, ^22^ Results from this analysis determined the subtype and gene‐specific reverse transcriptase (RT)‐PCR and nested PCR primer sets for amplification of NS3/4A and NS5A genes from baseline samples. Phylogenetic analyses were subsequently conducted using HCV NS3 (1‐543 nt) and NS5A (1‐645 nt) nucleotide sequences from baseline samples. Nucleotide sequences for NS3, NS5A, and NS5B were aligned using the MAFFT sequence alignment method.^23^ Phylogenetic trees were constructed using the neighbor‐joining tree‐building method^24^, ^25^ with the HKY85 nucleotide substitution model^26^ and 1000 bootstrapping replicates. The final HCV GT2 subtype assignment was determined by consensus between NS3, NS5A, and NS5B results.^27^

### 2.4 Patient sample processing and sequence analysis

Viral RNA isolation, RT‐PCR, and nested PCR were conducted for the target genes NS3/4A and NS5A on samples with HCV RNA ≥1000 IU/mL, as previously described.^19^, ^28^, ^29^ For patients who achieved SVR, population nucleotide sequencing was conducted on the regions encoding HCV NS3 amino acids 1‐181 and NS5A amino acids 1‐215 from all available baseline samples. In patients who experienced VF, population sequencing was conducted for the regions encoding full‐length NS3/4A (amino acids 1‐685) and NS5A (amino acids 1‐466) at baseline, the time of failure, post‐treatment week 24, and post‐treatment week 48. Clonal sequencing was conducted on post‐treatment samples from patients who experienced VF if substitutions at signature amino acid positions were not detected within a target by population sequencing. For clonal sequencing, the nested gel‐purified NS3 or NS5A PCR products were inserted into pJET1.2/blunt Cloning Vector using CloneJET PCR Cloning Kit (Thermo Scientific, Pittsburgh, PA), and >80 individual colonies were sequenced for each sample for the regions encoding NS3 amino acids 1‐181 or NS5A amino acids 1‐215.^30^

The following positions are considered signature amino acid positions for the protease and NS5A inhibitor class in HCV GT2: 56, 155, 156, and 168 in NS3; 24, 28, 30, 31, 32, 58, 92, and 93 in NS5A. Baseline sequences were compared to prototypic reference sequence 2a JFH‐1 (GenBank accession number AB047639) for GT2a sequences, and prototypic reference sequence 2b HC‐J8 (GenBank accession number D10988) for GT2b sequences. Fisher's exact test with a two‐sided significance level of 0.05 was used to compare the impact of baseline polymorphisms in NS3 and NS5A on treatment outcome. Treatment‐emergent amino acid substitutions were identified by comparing translated post‐baseline sequences to the respective baseline sequence. Substitutions by clonal sequencing are defined as substitutions observed in two or more clones (out of at least 80 clones) from a sample obtained at a post‐baseline time point relative to the reference sequence.

### 2.5 Stable HCV replicon cell lines

The non‐chimeric GT2a JFH‐1 (GenBank accession number AB047639) replicon was bicistronic, and the first cistron contained the 5′ nontranslated region (NTR) from GT2a JFH‐1 followed by a firefly luciferase reporter gene and the neomycin phosphotransferase (Neo) gene. This was followed by the EMCV IRES and the second cistron containing the GT2a JFH‐1 NS3‐NS5B coding region, and 3′ NTR derived from GT2a JFH‐1. The NS3 GT2b chimeric stable replicon was generated by insertion of the region encoding the first 251 amino acids of NS3 from a synthetically constructed GT2b protease domain in place of the corresponding region from 2a‐JFH‐1. The GT2b NS3 gene sequence was synthetically constructed based on the generation of a consensus sequence derived from the alignment of 15 GT2b sequences. The stable cell lines were generated by introducing this construct into Huh‐7 human hepatoma cells, and the cell lines were maintained in Dulbecco's modified Eagle medium containing 200 μg/mL G418 (both from Invitrogen, Carlsbad, CA) and 10% (v/v) fetal bovine serum (Atlanta Biologicals, Flowery Branch, GA).

### 2.6 Antiviral activity of NS3 or NS5A amino acid substitutions in transient HCV replicons

The non‐chimeric NS3 GT2a JFH‐1 transient replicon and the chimeric GT2b NS3 transient replicon both contained the same construct described above for the stable cell lines, except that the transient replicons lacked the Neo gene. The wild‐type GT2a non‐chimeric or 2b chimeric replicon sequence for NS3 matched the 2a JFH‐1 or 2b HC‐J8 reference amino acid sequence at all signature amino acid positions in NS3.

The generation and sequence of the chimeric HCV GT1b Con1 (GenBank accession number AJ238799) subgenomic, transient replicons for NS5A GT2a and 2b were previously described.^19^ The NS5A regions for the generation of the wild‐type chimeric transient replicons were derived from GT2a or 2b clinical isolates. The wild‐type GT2a or 2b chimeric replicon sequence for NS5A matched the 2a JFH‐1 or 2b HC‐J8 reference amino acid sequence at all signature positions except for amino acid position 31 in NS5A. The 2a wild‐type chimeric transient replicon has M31 in NS5A, and the 2b wild‐type chimeric transient replicon has L31 in NS5A. Some of the NS5A amino acid substitutions were evaluated in the background of L31 as well as M31 to completely assess ombitasvir activity.

HCV NS3 or NS5A amino acid substitutions were identified either during in vitro selection experiments in cell culture, or in patients who experienced VF in M12‐536 or GIFT‐II. Amino acid substitutions in NS3 or NS5A were synthesized (Integrated DNA Technologies, Coralville, IA) and inserted into the NS3 or NS5A GT2a or 2b replicon shuttle‐vector constructs. For the transient replicon assay, subgenomic replicon RNA was generated by plasmid DNA linearization followed by in vitro RNA transcription. The chimeric replicon RNA was transfected via electroporation into a Huh‐7 derived cell line, and luciferase expression (Promega, Madison, WI) was measured 4 days post‐transfection to determine the inhibitory effect of ombitasvir or paritaprevir on HCV chimeric replicon replication.^31^, ^32^ Additional details regarding assay controls and methodology can be found in reference.^31^ Due to the high replication capacity of the GT2a JFH‐1 replicon,^33^ the EC_50_ values of paritaprevir against GT2 NS3 substitutions were generated using a reduced amount of transfected RNA in order to stay within the linear range of the luciferase assay. The EC_50_ values for NS3 and NS5A were calculated using a nonlinear regression curve fitting to the 4‐parameter logistic equation in the Prism 4/5 software (GraphPad Software, Inc., La Jolla, CA). Mean EC_50_ values and standard deviation were calculated from at least three independent experiments.

## 3 RESULTS

### 3.1 HCV GT2 subtype prevalence and SVR rates in studies M12‐536 and GIFT‐II

HCV subtype was determined by phylogenetic analysis for 37 GT2‐infected patients enrolled in phase 2 study M12‐536, and 170 GT2‐infected patients enrolled in phase 3 study GIFT‐II. The comparison between genotype and subtype determined by the Versant HCV Genotype Inno‐LiPA Assay v2.0 and phylogenetic analysis is shown in Supplementary Table S1. The overall prevalence of GT2a and GT2b infection by phylogenetic analysis was 60.4% (125/207) and 38.2% (79/207), respectively, with similar subtype frequency detected in each study. The SVR rates by GT2 subtype and study subgroup are shown in Table 1 for HCV‐infected Japanese patients enrolled in clinical studies M12‐536 and GIFT‐II. In both studies, patients with HCV GT2a infection achieved higher SVR rates than patients with GT2b infection for all subgroups analyzed, including treatment dose and duration, prior treatment experience, and cirrhosis status.^7^, ^20^

**Table 1:** SVR rates by HCV GT2 subtype in Japanese studies M12‐536 and GIFT‐II

Study	Subgroup	Subtype	SVR rate, % (n/N)^a^	Number of VFs^b^
M12‐536^c^	25/100/100 mg	2a	81.8 (9/11)	2
		2b	14.3 (1/7)	6
	25/150/100 mg	2a	100 (9/9)	0
		2b	37.5 (3/8)	5
GIFT‐II^d^	12 wk, TN, non‐cirrhotic	2a	82.8 (24/29)	3
		2b	63.2 (12/19)	7^e^
	12 wk, TE, non‐cirrhotic	2a	86.4 (19/22)	3
		2b	22.2 (2/9)	7
	12 wk, cirrhotic	2a	100 (3/3)	0
		2b	50 (1/2)	1
	16 wk, TN, non‐cirrhotic	2a	93.9 (31/33)	2
		2b	85.7 (12/14)	2
	16 wk, TE, non‐cirrhotic	2a	93.8 (15/16)	1
		2b	56.3 (9/16)	6
	16 wk, cirrhotic	2a	50 (1/2)	0
		2b	25 (1/4)	3

SVR, sustained virologic response; TN, Treatment‐naïve; TE, Treatment‐experienced to an IFN‐containing regimen with or without RBV; VF, virologic failure.

^a^ % of patients achieving SVR_12_ in study GIFT‐II,^20^ or SVR_24_ in study M12‐536.^7^

^b^ Number of patients who experienced virologic failure. Excludes patients who did not achieve SVR due to non‐virologic reasons such as premature study drug discontinuation or missing SVR_12_ or SVR_24_ data.

^c^ Patients in study M12‐536 received OBV/PTV/r for 12 weeks, and all patients were treatment‐experienced to pegIFN/RBV and were non‐cirrhotic.

^d^ GIFT‐II treatment regimen included co‐formulated ombitasvir/paritaprevir/r (25/150/100 mg QD) + RBV for 12 or 16 weeks. Patients were treatment naïve or treatment‐experienced to an IFN containing regimen (IFN alpha, beta, or pegIFN) with or without RBV.

^e^ One GT2b‐infected treatment‐naïve patient achieved SVR_12_ but relapsed at PTW24.

### 3.2 Lack of impact of baseline polymorphisms in NS3 and NS5A on SVR

NS3 D168E in GT2a, which confers 5.3‐fold resistance to paritaprevir, was detected in 1 patient; resistance‐conferring baseline polymorphisms were not detected in GT2b (Table 2). Baseline polymorphisms in NS5A that confer resistance to ombitasvir in GT2a (T24A/S, F28C, L/M31I, and C92S) were detected at a frequency of 11.2% (14/125). Baseline polymorphisms in NS5A that confer resistance to ombitasvir in GT2b (L28F, L/M31I, and C92S) were detected in 14.1% (11/78) of the patients (Table 2).

**Table 2:** Prevalence of baseline polymorphisms in NS3 and NS5A in HCV GT2a and 2b‐infected patients

GT2 subtype	Target	Baseline polymorphism	Prevalence % (n/N)^a^
GT 2a	NS3	Any^b^	2.5 (3/122)
		Y56F	1.6 (2/122)
		D168E^c^	0.8 (1/122)
		PTV‐specific^c^	0.8 (1/122)
GT 2a	NS5A	Any^b^	95.2 (119/125)
		T24A^d^	8.0 (10/125)
		T24S^d^	2.4 (3/125)
		F28C^d^/L	2.4 (3/125)
		K30R/T	2.4 (3/125)
		L31I^d^	0.8 (1/125)
		L31M	92.8 (116/125)
		P58H/S	3.2 (4/125)
		C92S^d^	1.6 (2/125)
		OBV‐specific^d^	11.2 (14/125)
GT 2b	NS3	Any^b^	9.0 (7/78)
		Y56F/H	9.0 (7/78)
		D168V^c^	(0/78)
		PTV‐specific^c^	(0/78)
GT 2b	NS5A	Any^b^	41.0 (32/78)
		L28F^d^	10.3 (8/78)
		K30R	3.8 (3/78)
		M31I^d^	1.3 (1/78)
		M31L	21.8 (17/78)
		P58S/T	3.8 (3/78)
		C92S^d^/W	3.8 (3/78)
		OBV‐specific^d^	14.1 (11/78)

^a^ % of patients with the polymorphism at the corresponding amino acid position, *n* = number of patients with baseline polymorphism, *N* = total number of samples sequenced.

^b^ Number of patients with any baseline polymorphism in the designated target at signature resistance‐associated amino acid positions.

^c^ Baseline polymorphism detected in NS3 that confers resistance to paritaprevir.

^d^ Baseline polymorphism detected in NS5A that confers resistance to ombitasvir. Patient sequences with single or multiple OBV‐specific resistance‐conferring polymorphisms were included in the total count, and sequences were not double counted.

The impact of baseline polymorphisms in NS3 and NS5A on treatment outcome was evaluated for studies M12‐536 (Supplementary Table S2) and GIFT‐II (Table 3) by comparing SVR rates in patients with and without baseline polymorphisms. In study M12‐536, SVR_24_ rates were similar in patients with or without an NS3 or NS5A polymorphism at baseline with GT2a or GT2b infection (Supplementary Table S2). In GIFT‐II, SVR_12_ rates were compared separately by GT2 subtype for treatment‐naïve and IFN treatment‐experienced patients without cirrhosis, and for patients with cirrhosis, who received 12 or 16 weeks of treatment. For GT2a infection, the SVR_12_ rates were similar in non‐cirrhotic treatment‐naïve and treatment‐experienced patients with or without an NS3 or NS5A polymorphism at baseline (Table 3). The treatment‐experienced GT2a‐infected patient with D168E in NS3 at baseline achieved SVR_12_ in the 16 week Arm. Twelve out of 13 GT2a‐infected patients (92.3%) with baseline polymorphisms in NS5A that confer resistance to ombitasvir in GT2a (T24A/S, F28C, L/M31I, and C92S) achieved SVR_12_, including one patient with double substitution T24A + F28C in NS5A. The one patient with baseline polymorphisms that did not achieve SVR_12_ had T24A + L31M + C92S in NS5A at baseline. There was no significant difference in SVR_12_ rates for GT2a‐infected patients with M or L amino acids at position 31 in NS5A.

**Table 3:** Impact of baseline polymorphisms in NS3 and NS5A on treatment outcome in study GIFT‐II for Japanese GT2‐infected patients without cirrhosis

		SVR_12_ rate % (n/N)^a^					
		Treatment‐naive		Treatment‐experienced		Total	
GT	BP	With BP	Without BP	With BP	Without BP	With BP	Without BP
12 weeks							
GT2a^b^, NS5A	T24A/S	100 (4/4)	87 (20/23)	(0/1)	90 (19/21)	80 (4/5)	89 (39/44)
	F28L	100 (1/1)	88 (23/26)	‐	86 (19/22)	100 (1/1)	88 (42/48)
	L31M	88 (22/25)	100 (2/2)	86 (19/22)	‐	87 (41/47)	100 (2/2)
	P58S	100 (1/1)	88 (23/26)	100 (1/1)	86 (18/21)	100 (2/2)	87 (41/47)
	C92S	‐	89 (24/27)	50 (1/2)	90 (18/20)	50 (1/2)	89 (42/47)
GT2b, NS3	Y56F/H	100 (1/1)	65 (11/17)	(0/1)	25 (2/8)	50 (1/2)	52 (13/25)
GT2b, NS5A	L28F	67 (2/3)	67 (10/15)	50 (1/2)	14 (1/7)	60 (3/5)	50 (11/22)
	K30R	100 (1/1)	65 (11/17)	‐	22 (2/9)	100 (1/1)	50 (13/26)
	M31L	83 (5/6)	58 (7/12)	(0/1)	25 (2/8)	71 (5/7)	45 (9/20)
	P58S	100 (1/1)	65 (11/17)	‐	22 (2/9)	100 (1/1)	50 (13/26)
	C92S	67 (2/3)	67 (10/15)	‐	22 (2/9)	67 (2/3)	50 (12/24)
16 weeks							
GT2a, NS3	Y56F	‐	94 (30/32)	(0/1)	100 (15/15)	(0/1)	96 (45/47)
	D168E	‐	94 (30/32)	100 (1/1)	93 (14/15)	100 (1/1)	94 (44/47)
GT2a, NS5A	T24A/S	100 (5/5)	93 (26/28)	100 (1/1)	93 (14/15)	100 (6/6)	93 (40/43)
	F28C/L	100 (2/2)	94 (29/31)	‐	94 (15/16)	100 (2/2)	94 (44/47)
	K30R	100 (1/1)	94 (30/32)	‐	94 (15/16)	100 (1/1)	94 (45/48)
	L31I	‐	94 (31/33)	100 (1/1)	93 (14/15)	100 (1/1)	94 (45/48)
	L31M	93 (28/30)	100 (3/3)	93 (13/14)	100 (2/2)	93 (41/44)	100 (5/5)
	P58H	100 (1/1)	94 (30/32)	‐	94 (15/16)	100 (1/1)	94 (45/48)
GT2b, NS3	Y56F	‐	86 (12/14)	(0/1)	69 (9/13)	(0/1)	78 (21/27)
GT2b, NS5A	L28F	100 (1/1)	83 (10/12)	(0/1)	64 (9/14)	50 (1/2)	73 (19/26)
	K30R	‐	85 (11/13)	100 (1/1)	57 (8/14)	100 (1/1)	70 (19/27)
	M31I	100 (1/1)	83 (10/12)	‐	60 (9/15)	100 (1/1)	70 (19/27)
	M31L	100 (2/2)	82 (9/11)	‐	60 (9/15)	100 (2/2)	69 (18/26)
	P58S/T	50 (1/2)	91 (10/11)	‐	60 (9/15)	50 (1/2)	73 (19/26)

GT, genotype; BP, baseline polymorphism; TN, treatment‐naïve; TE, treatment‐experienced to an IFN‐containing regimen (IFN alpha, beta, or pegIFN) with or without RBV.

^a^ % of patients achieving SVR_12_ with or without the polymorphism at the corresponding amino acid position, *n* = number of patients with baseline polymorphism, *N* = total number of samples sequenced. Patients with compensated cirrhosis and patients not achieving SVR_12_ for reasons other than VF were excluded from this analysis.

^b^ Baseline polymorphisms were not detected in NS3.

Although there was an increased rate of virologic failure in patients with HCV GT2b infection in study GIFT‐II, SVR_12_ rates were similar in GT2b‐infected patients with or without an NS3 or NS5A polymorphism at baseline (Table 3). Overall SVR_12_ rates were higher for treatment‐naïve compared to pegIFN/RBV treatment‐experienced GT2b‐infected patients without cirrhosis (Table 1). NS3 polymorphisms conferring resistance to paritaprevir were not detected in any of the non‐cirrhotic or cirrhotic GT2b‐infected patients at baseline. Seven out of 10 (70%) GT2b‐infected patients without cirrhosis who had NS5A baseline polymorphisms that confer resistance to ombitasvir in GT2b (L28F, L/M31I, and C92S) achieved SVR_12_. The presence of M or L amino acids at position 31 in NS5A was not associated with differences in treatment outcome in GT2b‐infected patients without cirrhosis or with compensated cirrhosis.

### 3.3 Treatment‐emergent substitutions in patients not achieving SVR

In phase 2 study M12‐536, two patients with HCV GT2a infection who received the 25/100/100 mg dose of OBV/PTV/r experienced virologic failure (Table 4). Treatment‐emergent amino acid substitutions were detected at position D168 in NS3 in both GT2a‐infected patients at the time of VF, but no treatment‐emergent substitutions were detected in NS5A at the time of VF. Eleven patients with GT2b infection experienced VF. Treatment‐emergent substitutions at D168 were detected in NS3 at the time of VF in 100% (11/11) of VF patients. Treatment‐emergent substitutions in NS5A were detected in 90.9% (10/11) of patients, the most common of which was L28F detected alone or in combination with another substitution in 73% (8/11) of patients (Table 4).

**Table 4:** Treatment‐emergent, resistance‐associated substitutions in patients who experienced virologic failure in studies M12‐536 and GIFT‐II

			M12‐536, n/N^a^		GIFT‐II, n/N^a^			
GT	Target	Treatment‐emergent substitution	25/100/100 mg^b^	25/150/100 mg^b^	12 wk, TN	12 wk, TE	16 wk, TN	16 wk, TE
2a	NS3	Any^c^	2/2	NA	1/3	1/3	2/2	1/1
		Y56H, D168V	1/2			1/3		
		D168A/E/Y	1/2		1/3		2/2	1/1
2a	NS5A	Any^c^	0/2	NA	2/3	0/3	2/2	1/1
		F28S			1/3		2/2	
		K30M			1/3			
		T24A, L31I/V						1/1
2a	NS3 + NS5A	Any^c^	0/2	NA	1/3	0/3	2/2	1/1
2b	NS3	Any^c^	6/6	5/5	5/7	8/8	2/2	9/9
		D168A/F/H/L/N/P/S/T/V/Y	6/6	5/5	5/7	8/8	2/2	9/9
2b	NS5A	Any^c^	6/6	4/5	6/7	7/8	2/2	8/9
		L28F	3/6	1/5	4/7	3/8		4/9
		M31V		1/5		1/8		
		C92Y				1/8		
		Y93H	1/6		1/7			
		L28F, C92S/Y	1/6	1/5		1/8		
		L28F, M31I		1/5			2/2	3/9
		L28F, M31V	1/6			1/8		
		M31V, C92S			1/7			
		M31V, Y93H						1/9
2b	NS3 + NS5A	Any^c^	6/6	4/5	4/7	7/8	2/2	8/9

GT, genotype; NA, not applicable as there were no GT2a VFs in the study arm; TN, treatment‐naïve; TE, treatment‐experienced to an IFN‐containing regimen (IFN alpha, beta, or pegIFN) with or without RBV.

^a^ *n* = number of patients with the treatment‐emergent substitution, *N* = total number of patients who experienced VF in the designated study Arm. Patients without cirrhosis or with compensated cirrhosis who experienced VF are included in the analysis.

^b^ Patients in study M12‐536 received OBV/PTV/r for 12 weeks, and all patients were treatment‐experienced to pegIFN/RBV.

^c^ Number of patients with any treatment‐emergent substitution at signature resistance‐associated amino acid positions in the designated target.

Nine GT2a‐infected patients in study GIFT‐II experienced VF (Tables 1 and 4), including 6 patients in the 12 week Arm and 3 patients in the 16 week Arm. Treatment‐emergent substitutions in NS3 were not detected in 44.4% (4/9) of GT2a‐infected patients at the time of VF, while amino acid substitutions at position D168 in NS3 were detected at the time of VF in the remaining 5 patients. In NS5A, treatment‐emergent substitutions were detected in five out of nine patients at the time of VF, the most common of which was F28S (3/9). Three patients had no substitutions detected in NS5A at baseline or at the time of VF, while the one patient who had T24A + L31M + C92S detected at baseline in NS5A had the same polymorphisms present at the time of VF. The absence of treatment‐emergent substitutions was confirmed by clonal sequencing, which has an approximate sensitivity threshold of 5% for detection of minor HCV variants.

Among GT2b‐infected patients in study GIFT‐II, 26 experienced virologic failure (Tables 1 and 4). Treatment‐emergent substitutions in NS3 were detected at amino acid position D168 in 92.3% (24/26) of patients at the time of VF (Table 4). NS3 variants were not detected at the time of VF in 2 treatment‐naïve patients who received 12 weeks of treatment. In NS5A, treatment‐emergent substitutions were detected in 88.5% (23/26) of patients at the time of VF, while 3 patients had NS5A variant L28F present at baseline and at the time of VF. The most common treatment‐emergent substitution detected at the time of VF in NS5A was L28F (17/26) alone or in combination with another substitution in NS5A. There was no significant difference in the distribution of substitutions in NS3 at position D168, or NS5A substitutions L28F, L/M31V, C92S/Y, or Y93H detected at the time of VF between GT 2b‐infected patients who received 12 or 16 weeks of treatment. Similar NS3 or NS5A substitutions were detected at the time of VF between GT2b‐infected treatment‐naïve and treatment‐experienced patients.

Treatment‐emergent substitutions seen at the time of VF in studies M12‐536 or GIFT‐II were evaluated for resistance to paritaprevir or ombitasvir in HCV transient replicon assays (Table 5). Ombitasvir displayed potent antiviral activity against the NS5A wild‐type 2a chimeric transient replicon with M31 (mean EC_50_ = 1.3 pM) and the NS5A 2a JFH‐1 chimeric transient replicon with L31 (mean EC_50_ = 1.9 pM). In the NS5A wild‐type 2b chimeric replicon, ombitasvir displayed similar antiviral activity against GT2b NS5A with the polymorphism L31 (mean EC_50_ = 0.71 pM) or M31 (mean EC_50_ = 1.1 pM). Some NS5A substitutions were evaluated in combination with both L31 and M31, as shown in Table 5.

**Table 5:** In vitro activity of paritaprevir or ombitasvir against HCV GT2 transient subgenomic replicons containing amino acid substitutions in NS3 or NS5A

Replicon subtype	NS3 substitutions	Paritaprevir mean EC_50_ ± SD, nM	Fold resistance to paritaprevir^c^	NS5A substitutions	Ombitasvir mean EC_50_ ± SD, pM	Fold resistance to ombitasvir^c^
2a	Wild‐type^a^	17 ± 2.0	‐	Wild‐type^b^	1.3 ± 0.1	‐
	Y56F	8.6 ± 1.9	0.5	T24A	50 ± 6.9	38
	Y56H	64 ± 17	3.8	T24S	87 ± 38	67
	D168A	306 ± 34	18	F28C	652 ± 101	501
	D168E	89 ± 15	5.3	F28S	15 103 ± 1760	11 618
	D168H	213 ± 4.4	13	K30M	0.23 ± 0.04	0.2
	D168V	228 ± 32	13	M31I	25 ± 5.9	19
	D168Y	222 ± 46	13	M31V	ND^d^	‐
	Y56H + D168V	443 ± 48	26	C92S	16 ± 4.2	13
				Y93H	ND^d^	‐
				T24A + M31L	20 ± 0.33	15
				T24A + C92S	1250 ± 278	962
				T24S + F28C	6026 ± 1538	4636
				F28S + Y93H	ND^d^	‐
2b	Wild‐type^a^	114 ± 27	‐	Wild‐type^b^	0.71 ± 0.21	‐
	Y56F	67 ± 4.1	0.6	L28F	33 ± 6.1	47
	D168A	1309 ± 276	11	L31I	20 ± 3.3	28
	D168E	256 ± 58	2.2	L31M	1.1 ± 0.12	1.5
	D168F	1239 ± 182	11	L31V	361 ± 70	511
	D168H	1055 ± 54	9.3	C92S	5.7 ± 0.85	8
	D168S	1008 ± 301	8.8	C92Y	7.8 ± 2.1	11
	D168T	1165 ± 99	10	Y93H	ND^d^	‐
	D168V	1073 ± 174	9.4	Y93N	ND^d^	‐
	D168Y	803 ± 138	7.0	L28F + L31I	1013 ± 143	1427
	Y56F + D168A	1381 ± 165	12	L28F + L31M	176 ± 30	247
	Y56F + D168E	62 ± 5.7	0.5	L28F + L31V	ND^d^	‐
	Y56F + D168V	1102 ± 143	9.7	L31M + C92S	27 ± 5.1	38
	Y56H + D168E	310 ± 36	2.7	L31M + C92Y	16 ± 1.5	23
				L31V + C92S	5271 ± 476	7423
				L28F + L31M + C92S	1482 ± 105	2088
				L28F + L31M + C92Y	ND^d^	‐

^a^ The wild‐type GT2a non‐chimeric or 2b chimeric replicon sequence for NS3 matched the 2a JFH‐1 or 2b HC‐J8 reference amino acid sequence, respectively, at all NS3 signature resistance‐associated amino acid positions.

^b^ The wild‐type GT2a or 2b chimeric replicon sequence for NS5A matched the 2a JFH‐1 or 2b HC‐J8 reference amino acid sequence at signature resistance‐associated amino acid positions, except for amino acid position 31 in NS5A. The NS5A 2a wild‐type chimeric transient replicon had M31, and the NS5A 2b wild‐type chimeric transient replicon had L31.

^c^ Substitution EC_50_/Wild‐Type EC_50_.

^d^ EC_50_ ± SD could not be determined due to the poor replication capacity of the chimeric replicon containing the amino acid substitution.

### 3.4 Persistence of treatment‐emergent substitutions through post‐treatment week 48

**Figure 1:**
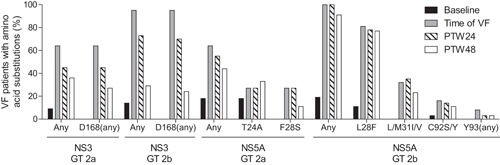
Persistence of resistance‐associated baseline polymorphisms and treatment‐emergent substitutions in GT 2a or 2b‐infected patients who experienced virologic failure. The percentage of VF patients with the designated baseline or treatment‐emergent substitution is shown for the baseline, time of VF, post‐treatment week 24, and post‐treatment week 48 time points. Columns denoted as “Any” include patients with any baseline polymorphism or treatment‐emergent substitution for NS3 or NS5A at signature resistance‐associated amino acid positions. Baseline polymorphisms L/M31 in NS5A were not included in the total “Any” count. Specific amino acid substitutions designated as “(any)” include any amino acid change from wild‐type in the total count

Baseline polymorphisms and treatment‐emergent substitutions in NS3 and NS5A were monitored for persistence through post‐treatment week 48 by population and clonal sequencing analyses (Figure 1). Among patients with HCV GT2a infection who experienced VF in studies M12‐536 or GIFT‐II, treatment‐emergent NS3 substitutions at amino acid position D168 remained detectable in 71.4% (5/7) of patients and 42.9% (3/7) of patients at post‐treatment week 24 and 48, respectively. In NS5A, treatment‐emergent substitutions were detected in 5 out of 11 VF patients, and these substitutions persisted through post‐treatment week 24 and 48 in 80% (4/5) and 40% (2/5) of patients, respectively. Overall, treatment‐emergent substitutions in NS3 and NS5A declined through post‐treatment week 48 in patients with GT2a infection.

Among patients with HCV GT2b infection who experienced VF in studies M12‐536 or GIFT‐II, treatment‐emergent substitutions at amino acid position D168 in NS3 remained detectable in 74.3% (26/35) and 25.0% (8/32) of patients with available sequence at post‐treatment week 24 and 48, respectively. Treatment‐emergent substitutions in NS5A were detected in 33 out of 37 GT2b‐infected patients at the time of VF, while the remaining 4 out of 37 patients had the NS5A L28F baseline polymorphism which persisted through post‐treatment week 48. Treatment‐emergent substitutions in NS5A persisted through post‐treatment week 24 and 48 in 100% (33/33) and 87.1% (27/31), respectively, of patients with available sequence. NS5A treatment‐emergent substitution L28F was detected in a majority of GT2b‐infected patients at the time of VF (81.2% [30/37]) and persisted through post‐treatment week 48. Overall, treatment‐emergent substitutions in patients with GT2b infection persisted in NS5A and declined in NS3 through post‐treatment week 48.

## 4 DISCUSSION

Japan has a high prevalence of GT2 infection (30%) among HCV‐infected patients. Clinical studies M12‐536 and GIFT‐II evaluated the efficacy and safety of OBV/PTV/r with or without RBV in HCV GT2‐infected Japanese patients, and the current report evaluated the viral characteristics of patient samples from these studies.

In GT2‐infected patients in studies M12‐536 and GIFT‐II, the prevalence of NS3 baseline polymorphisms that confer >5‐fold resistance to paritaprevir were rare (<1%). Baseline polymorphisms in NS5A that confer >5‐fold resistance to ombitasvir in GT2a (T24A/S, F28C, L/M31I, and C92S) were detected at a frequency of 11.2% (14/125) of the patient population. In GT2b, baseline polymorphisms in NS5A that confer >5‐fold resistance to ombitasvir (L28F, L/M31I, and C92S) were detected in 14.1% (11/78) of the patient population.

Among the 125 GT2a‐infected patients enrolled in studies M12‐536 and GIFT‐II, 11 patients experienced virologic failure. Treatment‐emergent substitutions in NS3 and NS5A declined through post‐treatment week 48 in patients with GT2a infection. All of the GT2a‐infected patients without treatment‐emergent substitutions detected in NS3 and NS5A received 12 weeks of treatment, indicating that 16 weeks of treatment may be required to fully suppress replication of wild‐type virus in HCV GT2a infection.

Among the GT2b‐infected patients, 37 out of 79 patients in M12‐536 and GIFT‐II experienced VF, including 31 patients with OTVF and 6 with relapse. Treatment‐emergent substitutions in GT2b‐infected patients persisted in NS5A and declined in NS3 through post‐treatment week 48. Although there was a higher rate of virologic failure in patients with GT2b infection, SVR_12_ rates were similar in patients with or without an NS5A baseline polymorphism that confers resistance to ombitasvir, indicating that the presence of baseline polymorphisms did not impact treatment outcome. In addition, logistic regression analysis in study GIFT‐II found that GT2a infection and a status of no prior treatment experience were associated with a higher odds of achieving SVR_12_,^20^ indicating that other factors such as prior treatment with pegIFN/RBV contributed to virologic failure. Additional viral or host factors, and differences in drug antiviral activity between GT2 subtypes, may have also contributed to the differential response seen between GT2a and GT2b‐infected patients. The EC_50_ value of paritaprevir is 11‐fold higher in GT2b compared to GT2a in stable replicon cell line assays (Supplementary Table S3). It is not clear whether or not the difference in paritaprevir in vitro activity between subtypes 2a and 2b plays a role in the higher rate of VF observed in HCV GT2b‐infected patients treated with OBV/PTV/r + RBV.

Currently, the approved IFN‐free DAA regimens in Japan for HCV GT2 infection are either sofosbuvir (SOF) with RBV for 12 weeks, or OBV/PTV/r plus RBV for 16 weeks. A recent resistance study reported that Japanese GT2‐infected patients who received SOF plus RBV and experienced relapse did not have NS5B resistance‐associated polymorphisms or substitutions detected at baseline or relapse, nor was there any change in SOF or RBV susceptibility in these patients.^34^ Our analysis examined the impact of baseline resistance‐associated polymorphisms in NS3 and NS5A for Japanese patients treated with OBV/PTV/r, and found no significant association of baseline polymorphisms with treatment outcome. The presence of methionine or leucine at position 31 in NS5A did not impact treatment outcome in either GT2a‐ or GT2b‐infected patients in GIFT‐II. Although there was an increased rate of virologic failure in patients with HCV GT2b infection, SVR_12_ rates were similar in GT2b‐infected patients with or without NS5A baseline polymorphisms that confer resistance to ombitasvir.

In summary, the lower SVR rate in GT2b‐infected patients was influenced by treatment duration and prior treatment with pegIFN/RBV,^20^ but not by baseline HCV polymorphisms. For the optimized treatment regimen of OBV/PTV/r + RBV for 16 weeks, the SVR_12_ rate for GT2a‐infected patients without cirrhosis was 93.9%, regardless of baseline viral polymorphisms and other patient characteristics. Baseline polymorphism testing is therefore not warranted as a pre‐screen for GT2‐infected patients seeking treatment with this regimen. The treatment regimen of OBV/PTV/r + RBV for 16 weeks is approved in Japan for non‐cirrhotic patients with HCV GT2 infection.

## Supporting information

Additional Supporting Information may be found online in the supporting information tab for this article.

**Table S1**. Comparison of HCV genotype and subtype between the LiPA 2.0 assay and phylogenetic analysis in M12‐536 and GIFT‐II.
**Table S2**. Impact of baseline polymorphisms in NS3 and NS5A on treatment outcome in study M12‐536 for Japanese GT2‐infected patients without cirrhosis.
**Table S3**. Activity of direct‐acting antivirals against HCV GT2 subgenomic replicon cells.
