# Supplementary material for: Resistance characterization of hepatitis C virus genotype 2 from Japanese patients treated with ombitasvir and paritaprevir/ritonavir
Source: J Med Virol. 2017 Sep 22;90(1):109–19. doi: 10.1002/jmv.24923 (PMC6680211; doi:10.1002/jmv.24923)
Supplement: Supplementary file 1 — Table S1. Comparison of HCV genotype and subtype between the LiPA 2.0 assay and phylogenetic analysis in M12‐536 and GIFT‐II. Table S2. Impact of baseline polymorphisms in NS3 and NS5A on treatment outcome in study M12‐536 for Japanese GT2‐infected patients without cirrhosis. Table S3. Activity of direct‐acting antivirals against HCV GT2 subgenomic replicon cells. [file JMV-90-109-s001.doc]

**Supplementary Material**

**Supplementary Table 1.** Comparison of HCV genotype and subtype between the LiPA 2.0 assay and phylogenetic analysis in M12-536 and GIFT-II.

|  | **Phylogenetic Analysis (n)** | | | |
| --- | --- | --- | --- | --- |
| **Versant HCV Genotype Inno-LiPA Assay v2.0** | **1a** | **1b** | **2a** | **2b** |
| 2 | 0 | 1 | 92 | 6 |
| 2a/2c | 0 | 0 | 33 | 0 |
| 2b | 1 | 1 | 0 | 73 |
| Total | 1 | 2 | 125 | 79 |

Supplementary Table 2. Impact of baseline polymorphisms in NS3 and NS5A on treatment outcome in study M12-536 for Japanese GT2-infected patients without cirrhosis.

|  |  | **25/100/100 mg**  **SVR_24_ rate % (n/N)^a^** | | | **25/150/100 mg**  **SVR_24_ rate % (n/N)^a^** | | |
| --- | --- | --- | --- | --- | --- | --- | --- |
| **Target and Subtype** | **Baseline**  **Polymorphism** | **With BP** | **Without BP** | **P-value** | **With BP** | **Without BP** | **P-value** |
| **GT 2a** |  |  |  |  |  |  |  |
| NS3 | Y56F | 100 (1/1) | 80 (8/10) | 1.0 | - | 100 (9/9) | - |
| NS5A | T24A | (0/1) | 90 (9/10) | 0.182 | - | 100 (9/9) | - |
|  | K30T | (0/1) | 90 (9/10) | 0.182 | 100 (1/1) | 100 (8/8) | 1.0 |
|  | L31M | 90 (9/10) | (0/1) | 0.182 | 100 (9/9) | - | - |
|  | P58S | - | 82 (9/11) | - | 100 (1/1) | 100 (8/8) | 1.0 |
| **GT 2b** |  |  |  |  |  |  |  |
| NS3 | Y56F | (0/1) | 17 (1/6) | 1.0 | 50 (1/2) | 33 (2/6) | 1.0 |
| NS5A | L28F | - | 14 (1/7) | - | (0/1) | 43 (3/7) | 1.0 |
|  | K30R | (0/1) | 17 (1/6) | 1.0 | - | 38 (3/8) | - |
|  | M31L | 100 (1/1) | (0/6) | 0.143 | 50 (1/2) | 33 (2/6) | 1.0 |

GT, genotype; BP, baseline polymorphism

1. % of patients achieving SVR_24_ with or without the polymorphism at the corresponding amino acid position, n = number of patients with baseline polymorphism, N = total number of samples sequenced. Patients not achieving SVR_24_ for reasons other than VF were excluded from the analysis. Patients received OBV/PTV/r for 12 weeks, and all patients were treatment-experienced to pegIFN/RBV.

**Supplementary Table 3. Activity of direct-acting antivirals against HCV GT2 subgenomic replicon cells.**

| **Target** | **Drug** | **HCV GT2 Replicon Subtype** | **Mean EC_50_ ± SD (nM)^a^** | **Reference^b^** |
| --- | --- | --- | --- | --- |
| NS3 | Paritaprevir | 2a JFH-1 | 9.8 **±** 1.5 | This publication |
|  | Paritaprevir | 2b | 107 ± 17 | This publication |
|  | Simeprevir | 2 | < 13^c^ | (1) |
|  | Grazoprevir | 2a JFH-1 | 8.0 ± 4 | (2) |
| NS5A | Ombitasvir | 2a JFH-1^e^ | 0.026 ± 0.001 | This publication |
|  | Ombitasvir | 2a-M31 | 0.012 ± 0.0027 | (3) |
|  | Ombitasvir | 2b-L31 | 0.0043 ± 0.0012 | (3) |
|  | Ombitasvir | 2b-M31 | 0.0011 ± 0.00012^d^ | This publication |
|  | Daclatasvir | 2a JFH-1 | 0.011 ± 0.001 | (4) |
|  | Daclatasvir | 2a-M31 | 13 ± 3.8 | (4) |
|  | Ledipasvir | 2a JFH-1 | 21 | (5) |
|  | Ledipasvir | 2a-M31 | 249^d^ | (5) |
|  | Ledipasvir | 2b-L31 | 16^d^ | (5) |
|  | Ledipasvir | 2b-M31 | 530^d^ | (5) |
|  | Elbasvir | 2a JFH-1 | 0.003 ± 0.001 | (6) |
|  | Elbasvir | 2b-M31 | 3.4 ± 2.6 | (6) |
| NS5B | Sofosbuvir | 2a JFH-1 | 37 ± 3.6^d^ | (7) |
|  | Sofosbuvir | 2b | 20 ± 4.4^d^ | (7) |

1. Mean EC_50_ ± standard deviation values are derived from stable subgenomic replicon cell lines unless otherwise noted.
2. (1) Lenz O, Verbinnen T, Lin TI, Vijgen L, Cummings MD, Lindberg J, et al. In vitro resistance profile of the hepatitis C virus NS3/4A protease inhibitor TMC435. Antimicrob Agents Chemother. 2010 May;54(5):1878-87.

(2) Summa V, Ludmerer SW, McCauley JA, Fandozzi C, Burlein C, Claudio G, et al. MK-5172, a selective inhibitor of hepatitis C virus NS3/4a protease with broad activity across genotypes and resistant variants. Antimicrob Agents Chemother. 2012 Aug;56(8):4161-7.

(3) Krishnan P, Beyer J, Mistry N, Koev G, Reisch T, DeGoey D, et al. In Vitro and In Vivo Antiviral Activity and Resistance Profile of Ombitasvir, an Inhibitor of Hepatitis C Virus NS5A. Antimicrob Agents Chemother. 2015 Feb;59(2):979-87.

(4) Wang C, Jia L, O'Boyle DR, 2nd, Sun JH, Rigat K, Valera L, et al. Comparison of daclatasvir resistance barriers on NS5A from hepatitis C virus genotypes 1 to 6: implications for cross-genotype activity. Antimicrob Agents Chemother. 2014 Sep;58(9):5155-63.

(5) Cheng G, Tian Y, Doehle B, Peng B, Corsa A, Lee YJ, et al. In Vitro Antiviral Activity and Resistance Profile Characterization of the Hepatitis C Virus NS5A Inhibitor Ledipasvir. Antimicrob Agents Chemother. 2016;60(3):1847-53.

(6) Coburn CA, Meinke PT, Chang W, Fandozzi CM, Graham DJ, Hu B, et al. Discovery of MK-8742: an HCV NS5A inhibitor with broad genotype activity. ChemMedChem. 2013 Dec;8(12):1930-40.

(7) Lam AM, Espiritu C, Bansal S, Micolochick Steuer HM, Niu C, Zennou V, et al. Genotype and subtype profiling of PSI-7977 as a nucleotide inhibitor of hepatitis C virus. Antimicrob Agents Chemother. 2012 Jun;56(6):3359-68.

1. Reported value is the half maximal inhibitory concentration (IC_50_) against purified NS3/4A enzyme.
2. Reported mean EC_50_ ± SD value from a transient replicon assay.
3. The 2a JFH-1 replicon contains L31 in NS5A.
